# Exogenous abscisic acid improves grain filling capacity under heat stress by enhancing antioxidative defense capability in rice

**DOI:** 10.1186/s12870-023-04638-5

**Published:** 2023-12-06

**Authors:** Xiaolong Liu, Xin Zhong, Jingpeng Liao, Ping Ji, Jinshuo Yang, Zhiruo Cao, Ximiao Duan, Junru Xiong, Ying Wang, Chen Xu, Hongtao Yang, Bo Peng, Kai Jiang

**Affiliations:** 1 College of Life Sciences and Resources and Environment, Yichun University, Jiangxi, 336000 Yichun China; 2 Engineering Technology Research Center of Jiangxi Universities and Colleges for Selenium Agriculture, Yichun University, Jiangxi, 336000 Yichun China; 3 Institute of Agricultural Resources and Environment, Jilin Academy of Agriculture Sciences, Jilin, 130033 Changchun China

**Keywords:** Abscisic acid (ABA), Heat stress, Priming Rice (*Oryza sativa* L.), Grain filling

## Abstract

**Background:**

Heat stress is a major restrictive factor that causes yield loss in rice. We previously reported the priming effect of abscisic acid (ABA) on rice for enhanced thermotolerance at the germination, seedling and heading stages. In the present study, we aimed to understand the priming effect and mechanism of ABA on grain filling capacity in rice under heat stress.

**Results:**

Rice plants were pretreated with distilled water, 50 μM ABA and 10 μM fluridone by leaf spraying at 8 d or 15 d after initial heading (AIH) stage and then were subjected to heat stress conditions of 38 °C day/30 °C night for 7 days, respectively. Exogenous ABA pretreatment significantly super-activated the ABA signaling pathway and improved the SOD, POD, CAT and APX enzyme activity levels, as well as upregulated the ROS-scavenging genes; and decreased the heat stress-induced ROS content (O_2_^–^ and H_2_O_2_) by 15.0–25.5% in rice grain under heat stress. ABA pretreatment also increased starch synthetase activities in rice grain under heat stress. Furthermore, ABA pretreatment significantly improved yield component indices and grain yield by 14.4–16.5% under heat stress. ABA pretreatment improved the milling quality and the quality of appearance and decreased the incidence of chalky kernels and chalkiness in rice grain and improved the rice grain cooking quality by improving starch content and gel consistence and decreasing the amylose percentage under heat stress. The application of paraquat caused overaccumulation of ROS, decreased starch synthetase activities and ultimately decreased starch content and grain yield. Exogenous antioxidants decreased ROS overaccumulation and increased starch content and grain yield under heat stress.

**Conclusion:**

Taken together, these results suggest that exogenous ABA has a potential priming effect for enhancing rice grain filling capacity under heat stress at grain filling stage mainly by inhibiting ROS overaccumulation and improving starch synthetase activities in rice grain.

**Supplementary Information:**

The online version contains supplementary material available at 10.1186/s12870-023-04638-5.

## Background

Heat stress, which is caused by extremely high temperatures or a lasting threshold of high-temperature weather, has become a severe restrictive factor for crop production worldwide [1]. Rice (*Oryza sativa* L.) is one of the most important food crops for the world’s population. Most of the rice growth period occurs in the long hot summer season, which results in severe heat stress for the growth and yield formation of rice [2, 3]. Previous study reported that the rice yield decreased by 3.2% due to a 1℃ increase in the mean temperature worldwide [4]. Thus, the demand for improving rice yield under heat stress conditions remains a major challenge for the expanding human population [5].

Heat stress leads to various types of damage to rice during the entire growth period. At the germination stage, the germination rate, bud length and radicle length have been found to be significantly decreased by heat stress at temperatures ≥ 35 ℃ [6]. Heat stress results in water loss, chlorophyll loss, photosystem structural damage, and membrane injury in rice seedlings, which can in turn lead to the withering and death of rice seedlings [7, 8]. Rice is more sensitive to heat stress during the reproductive growth stage. Heat stress causes a decrease in pollen viability and influences the meiosis process and development of young rice ears, which leads to a decrease in filled rice grain spikelets [9, 10]. The grain-filling stage is the critical period for starch synthesis, grain filling and yield formation in rice [5]. Heat stress at the grain-filling stage not only causes a decrease in yield component indices, but also causes a decline in the appearance, processing and cooking quality of rice [11, 12]. Previous study has reported that for rice quality outcomes, 8–21 d after initial heading is the vital stage of response to heat stress [13]. Thus, it is of vital importance to improve the thermotolerance in rice at grain-filling stage for the stable yield and quality under heat stress conditions.

Plants generate reactive oxygen species (ROS) that play important signaling roles in growth regulation in response to adverse stress conditions, such as occur under salt, alkaline, heat and chilling stress [14]. However, excess accumulation of ROS in plants results in damage to living cells, DNA, RNA and protein [15, 16]. Overaccumulation of ROS is an important injury mechanism by which heat stress inhibits plant growth [5]. Overaccumulation of superoxide anions (O_2_^–^) and hydrogen peroxide (H_2_O_2_) induced by heat stress in rice seeds is a vital limiting factor for the elongation of young buds and radicles [6]. Heat stress has been found to induce super-expression of the *respiratory burst oxidase homologue* (*Rboh*) family genes and to cause excess accumulation of ROS in rice seedlings, which led to severe membrane injury and even leaf withering in rice seedlings [8]. At the reproductive growth stage, the decline in pollen development and grain filling induced by heat stress was found to be associated with the overaccumulation of ROS in rice anthers, pollen and grains, causing a decline in the percentage of filled spikelets and in rice quality [10, 17, 18]. The improvement of antioxidative defense capability under heat stress conditions at the reproductive growth stage is a useful pathway for alleviating yield loss in plants [8, 19].

Phytohormones, such as abscisic acid (ABA), brassinosteroids (BRs), cytokinin (CK), salicylic acid (SA) and jasmonate (JA), act as an effective and efficient way for plants to cope with heat stress condition [20]. Among these phytohormones, ABA plays vital roles in enabling rice to adapt to heat stress [21]. The priming effect has been to be found to be an important promotion mechanism for ABA-enhanced stress tolerance in rice, conferring potential enhanced stress tolerance on seeds or seedlings pretreated with ABA [22]. The condition in plants pretreated with various natural compounds is called “primed”, and the natural compounds could be some organic compounds or phytohormone, such as hexanoic acid or abscisic acid [23]. Previous studies have reported that seedling pretreatment with ABA primes rice for enhanced alkaline stress tolerance and yield in saline-alkaline paddy soils [24, 25]. ABA also functions in the rice response to heat stress, and exogenous ABA improves thermotolerance and yield in rice under heat stress conditions [26, 27]. We previously reported on ABA primed rice for enhanced heat stress tolerance at the germination, seedling and heading stages, with the priming achieved by seed soaking, root drenching and leaf spraying [6, 8, 10]. Pretreatment with ABA has been shown to significantly strengthen the ABA signal in rice under heat stress and to improve the ROS-scavenging capability to inhibit the overaccumulation of ROS and mitigate membrane injury [6, 8, 10]. Furthermore, ABA pretreatment has been shown to superinduce the expression of stress tolerance-related genes in rice under heat stress, indicating that ABA has a potential priming effect on the gene expression regulation network in rice for coping with incoming stress factors [8, 10, 24]. However, the relationship between ROS and starch synthesis capacity and the effect of ABA on starch synthesis in rice under heat stress remain unknown.

This study aimed to gain insights into the effect and mechanism of ABA priming on starch synthesis capacity in rice under heat stress at the grain filling stage by focusing on the relationship between ROS accumulation and starch synthesis capacity. We hypothesized that exogenous ABA pretreatment has a potential priming effect that can improve starch synthesis capability by inhibiting ROS overaccumulation in rice grain under heat stress.

## Results

### ABA pretreatment strengthened the ABA signaling pathway in rice grain under heat stress

**Fig. 1:**
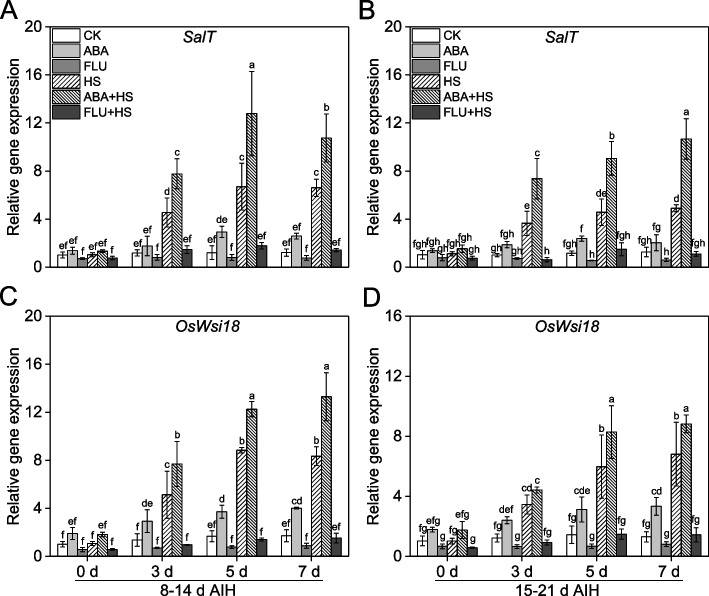
Exogenous ABA priming upregulated the transcriptional expression levels of ABA-responsive genes in rice grains under heat stress. The rice plants were sprayed with distilled water, 50 μM ABA or 10 μM fluridone (FLU) at the 5th d after initial heading stage (AIH) or 12th d after initial heading stage, and then subjected to unstressed or heat stress conditions at 8–14 days after initial heading (AIH) stage or 15–21 days after initial heading stage, respectively. Rice grains at the indicated treatment times of 0 d, 3 d, 5 d and 7 d were sampled in liquid nitrogen which were used to measure the expression levels of relative genes. Relative expression levels of ABA-responsive genes, *SalT* (**A**, **B**) and *OsWsi18* (**C**, **D**) were measured at the indicated treatment days. A quantitative real-time polymerase chain reaction was performed using *OsACT1* as an internal standard. The expression levels of the untreated control (CK) at 0 d were set as the unit to calculate the expression levels. Values are means ± SDs, *n* = *3*. Different letters on the column represent significant differences (*P* < *0.05*) between different treatments based on Duncan’s test

As shown in Fig. 1, the ABA signaling pathway was exactly activated by heat stress and ABA pretreatment as shown by the remarkable upregulation of two ABA-responsive genes, *SalT* and *OsWsi18* (Fig. 1). Under unstressed conditions, two ABA-responsive genes, *SalT* and *OsWsi18* was significantly upregulated by ABA pretreatment at 8–14 d and 15–21 d AIH stage, respectively. The expression levels of *SalT* and *OsWsi18* were increased by 32.4–142.8% and 72.8–155.4% at 8–14 d or 15–21 d AIH stage under unstressed conditions. Pretreatment with exogenous ABA significantly upregulated the expression of *SalT* and *OsWsi18* at 3, 5 and 7 d of heat stress at 8–14 d and 15–21 d AIH stage. Compared to HS treatment, the expression levels of *SalT* were increased by 62.8–90.8% and 97.8–117.7% with exogenous ABA pretreatment at 3, 5 and 7 d of heat stress at 8–14 d and 15–21 d AIH stage(Fig. 1A, B), and the expression levels of *OsWsi18* were increased by 38.7–59.3% and 27.9–38.6% by exogenous ABA pretreatment (Fig. 1C, D). While the expression levels of *SalT* and *OsWsi18* were significantly suppressed by exogenous FLU pretreatment in rice grain.

### ABA pretreatment decreased ROS accumulation and improved antioxidant defense capacity under heat stress

**Fig. 2:**
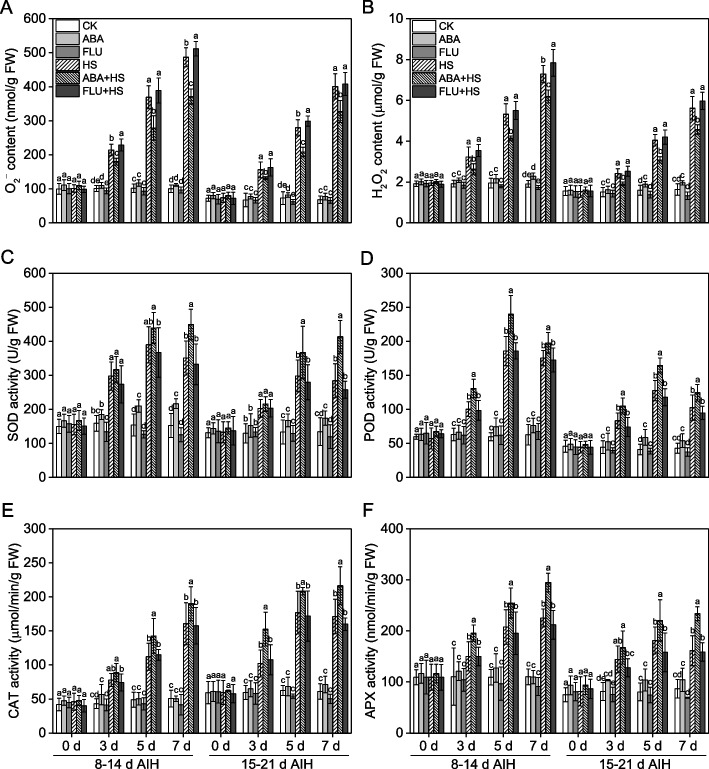
Exogenous ABA priming upregulated antioxidative defense capability in rice grains under heat stress. The rice plants were sprayed with distilled water, 50 μM ABA or 10 μM fluridone (FLU) at the 5th d after initial heading stage (AIH) or 12th d after initial heading stage, and then subjected to unstressed or heat stress conditions at 8–14 days after initial heading (AIH) stage or 15–21 days after initial heading stage, respectively. Rice grains at the indicated treatment times of 0 d, 3 d, 5 d and 7 d were sampled in liquid nitrogen which were used to measure the ROS contents and antioxidant enzyme activities. The accumulation of O_2_^–^ (**A**) and H_2_O_2_ (**B**) and the activity levels of SOD (**C**), POD (**D**), CAT (**E**), APX (**F**), in rice grains, were measured at the indicated treatment days. Values are means ± SDs, *n* = *3*. Different letters on the column represent significant differences (*P* < *0.05*) between different treatments at the same treatment day based on Duncan’s test

Under unstressed conditions, exogenous ABA pretreatment increased ROS content in rice grains. Pretreatment with exogenous ABA significantly inhibited ROS overaccumulation under heat stress as shown by the lower O_2_^–^ and H_2_O_2_ contents in rice grains (Fig. 2A, B). Compared to the HS treatment, exogenous ABA pretreatment decreased the O_2_^–^ content by 16.0–24.5% and 16.1–25.5% at 3, 5 and 7 d of heat stress at 8–14 d and 15–21 d AIH stage, and decreased the H_2_O_2_ content by 15.0–22.1% and 18.6–23.8% (Fig. 2A, B). While the levels of O_2_^–^ and H_2_O_2_ were increased by exogenous FLU pretreatment in rice grain under heat stress.

Exogenous ABA pretreatment also significantly improved the antioxidant defense capacity in rice grains as shown by the higher SOD, POD, CAT and APX activities with ABA pretreatment (Fig. 2C**-**F). Compared to the HS treatment, ABA pretreatment increased activity levels of SOD, POD, CAT and APX by 6.2–45.5%, 12.7–30.5%, 14.2–49.4% and 16.1–45.1% at 3, 5 and 7 d of heat stress at 8–14 d and 15–21 d AIH stage, respectively (Fig. 2C**-**F). The SOD activity in the HS treatment decreased at 7 d of heat stress, but it continuously increased with exogenous ABA pretreatment under heat stress at 8–14 d and 15–21 d AIH stage (Fig. 2C). Compared to the HS treatment, application of exogenous FLU decreased the antioxidant enzyme activity levels under heat stress.

We also analysed the relative expression levels of eight ROS-scavenging genes, as shown in Fig. S1. *OsCATA*, *OsCATB*, *OsAPX6*, *OsAPX7*, *OsCu/Zn-SOD*, *OsFeSOD*, *SodCc2* and *Putative copper/zinc superoxide dismutase* which encode CAT, APX and SOD, were significantly upregulated by heat stress. Furthermore, ABA pretreatment significantly upregulated the expression levels of *OsCATA*, *OsCATB*, *OsAPX6*, *OsAPX7*, *OsCu/Zn-SOD*, *OsFeSOD*, *SodCc2* and *Putative copper/zinc superoxide dismutase* in rice grains under heat stress (Fig. S1).

### ABA pretreatment improved starch synthetase activities in rice grain under heat stress

**Fig. 3:**
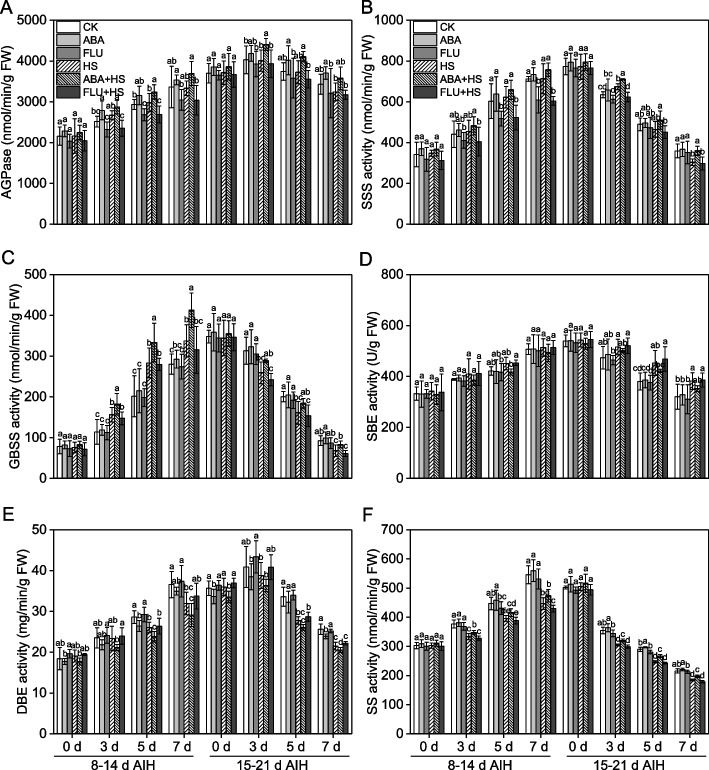
Exogenous ABA priming upregulated starch synthetase activities in rice grains under heat stress. The rice plants were sprayed with distilled water, 50 μM ABA or 10 μM fluridone (FLU) at the 5th d after initial heading stage (AIH) or 12th d after initial heading stage, and then subjected to unstressed or heat stress conditions at 8–14 days after initial heading (AIH) stage or 15–21 days after initial heading stage, respectively. Rice grains at the indicated treatment times of 0 d, 3 d, 5 d and 7 d were sampled in liquid nitrogen and used to measure the starch synthetase activities. Enzyme activity levels of (**A**) ADP-glucose pyrophosphorylase (AGPase), (**B**) soluble starch synthase (SSS), (**C**) granule-bound starch synthase (GBSS), (**D**) starch branching enzyme (SBE), (**E**) starch debranching enzyme (DBE), and (**F**) sucrose synthase (SS), in rice grains, were measured at the indicated treatment days. Values are means ± SDs, *n* = *3*. Different letters on the column represent significant differences (*P* < *0.05*) between different treatments at the same treatment day based on Duncan’s test

As shown in Fig. 3, the activity levels of AGPase, SSS, GBSS, SBE, DBE and SS all showed a single-peaked curve which that increased at the beginning and then decreased with the extension of the growth period. Under unstressed condition, compared to CK treatment, pretreatment with exogenous ABA for three days increased the AGPase, SSS, and GBSS activity levels and decreased the DBE activity at the gain filling stage.

Heat stress led to little effect to the activity of AGPase and SSS, but the activities of AGPase and SSS was significantly decreased by heat stress at 7 d of 8–14 d of AIH stage and 15–21 d of AIH stage (Fig. 3A, B). The GBSS activity levels showed an increasing tendency, compared to the CK treatment, at heat stress of 8–14 d of AIH stage, while it decreased compared to CK treatment at heat stress of 15–21 d of AIH stage (Fig. 3C). Heat stress induced increasing of SBE activity levels and decreasing of DBE activity levels at 15–21 d of AIH stage (Fig. 3D, E), indicating that the differences of amylose and amylopectin catabolism mechanism response to heat stress at grain filling stage, which needed further study. Heat stress led to decrease of SS activity levels in rice grain (Fig. 3F).

Under heat stress condition, exogenous ABA pretreatment increased the AGPase, SSS, GBSS, and SS activity levels and decreased the a SBE and DBE activity levels (Fig. 3). Compared to the HS treatment, the activity of AGPase was increased by 3.9–10.9% and 3.9–10.4%; the activity of SSS was increased by 5.7–6.2% and 3.0–18.6%; and the activity of GBSS was increased by 8.6–22.3% and 3.2–23.1%, at ABA + HS treatment at 8–14 d and 15–21 d AIH stage, respectively (Fig. 3A-C). Compared to the CK treatment, the increase amplitude of AGPase activity levels in ABA + HS treatment was higher than that in HS and FLU + HS treatment at 7 d into the period of 8–14 d AIH stage (Fig. 3A). However, compared to the HS treatment, the SBE and DBE activity levels were decreased by 2.7–7.7% and 4.4–9.5% at ABA + HS treatment at filling stage (Fig. 3D, E). Under heat stress condition, application of FLU decreased the activities of AGPase, SSS and GBSS, and increased activities of SBE and DBE, compared to ABA + HS treatment. Exogenous ABA pretreatment accelerated the decomposition of sucrose in rice grain under heat stress as shown by the increase of 2.7–8.2% in the SS activity at the grain filling stage (Fig. 3F).

### Exogenous ABA pretreatment increased grain yield under heat stress

As shown in Fig. S2, no apparent differences in plant growth indices, including shoot length, shoot dry weight, primary branches, secondary branches, and panicle length were observed with or without ABA pretreatment under unstressed or heat stress conditions. Heat stress led to a significant decrease in panicle weight, while exogenous ABA pretreatment increased panicle weight by 7.8% and 6.9% under heat stress at 8–14 d and 15–21 d AIH stage, respectively (Fig. S2).

**Fig. 4:**
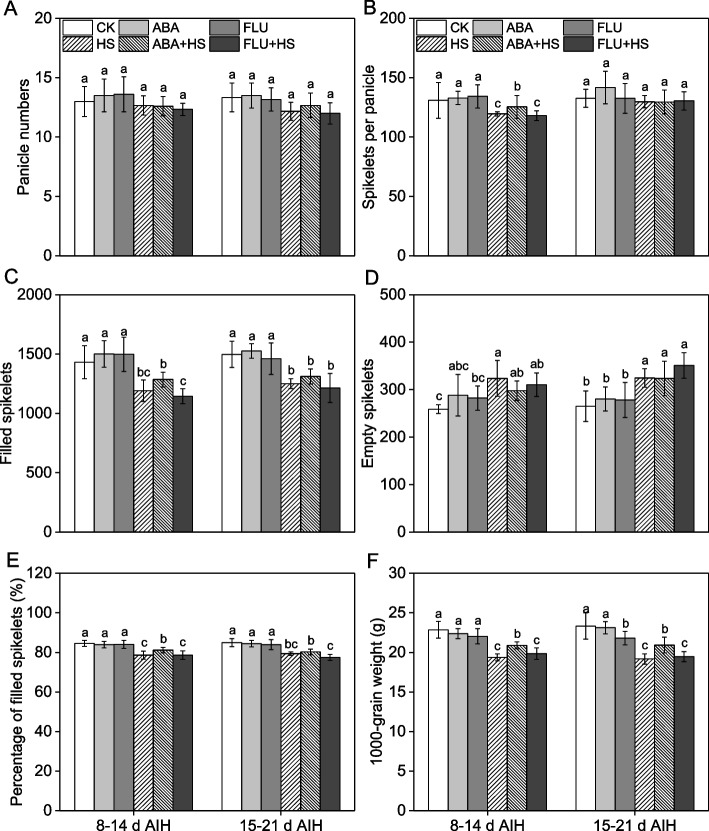
Exogenous ABA priming improved yield component indices in rice under heat stress. The rice plants were sprayed with distilled water, 50 μM ABA or 10 μM fluridone (FLU) at the 5th d after initial heading stage (AIH) or 12th d after initial heading stage, and then subjected to unstressed or heat stress conditions at 8–14 days after initial heading (AIH) stage or 15–21 days after initial heading stage, respectively. (**A**) Panicle number (PN), (**B**) spikelets per panicle (SP), (**C**) filled spikelets (FS), (**D**) empty spikelets (ES), (**E**) percentage of filled spikelets (PFS, %), and (**F**) 1000-grain weight (TGW, g) were measured at the mature stage. Values are means ± SDs, *n* = *3*. Different letters on the column represent significant differences (*P* < *0.05*) between different treatments based on Duncan’s test

As shown in Fig. 4, heat stress caused a significant decrease in spikelets per panicle (SP), filled spikelets (FS), percentage of filled spikelets (PFS), 1000-grain weight (TGW) and an increase in empty spikelets (ES) in rice at grain filling stage (Fig. 4). Exogenous ABA pretreatment significantly increased yield component indexes under heat stress as shown by FS, PFS, TGW and lower ES in rice grain (Fig. 4). Compared to the HS treatment, exogenous ABA pretreatment increased the SP by 4.9% under heat stress at 8–14 d AIH stage, while there was no significant differences between HS and ABA + HS treatment at 15–21 d AIH stage (Fig. 4B). The ES was significant decreased in ABA + HS treatment, compared to HS and FLU + HS treatment. However, significant differences of ES were in CK, ABA and FLU treatment under unstressed conditions (Fig. 4D), which may be due to the differences in expression levels of young panicle formation-related genes with different ABA signaling levels. Compared to the HS treatment, exogenous ABA pretreatment increased the FS, PFS and TGW by 7.9% and 4.8%, 3.2% and 1.0%, 7.8% and 9.2% under heat stress at 8–14 d and 15–21 d AIH stage, respectively (Fig. 4C, E, and F).

**Fig. 5:**
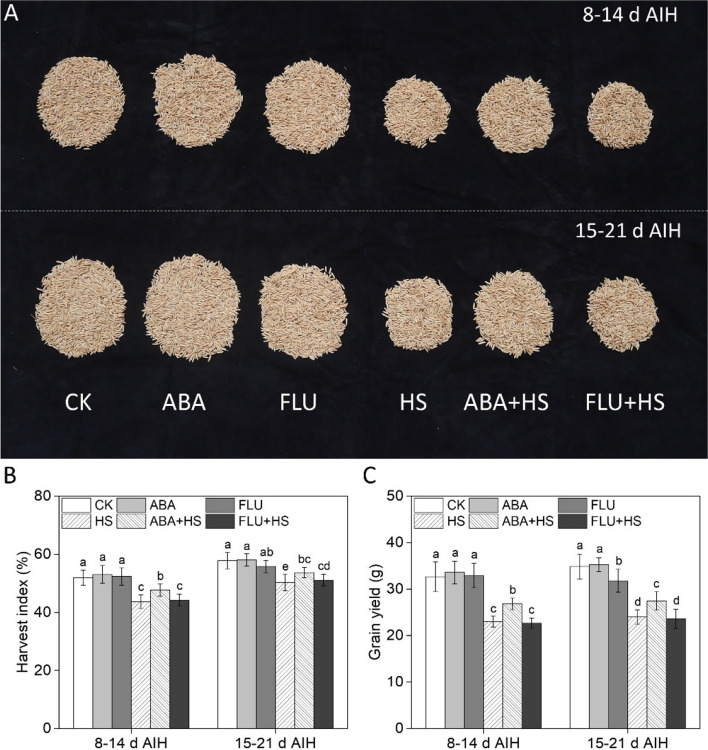
Exogenous ABA priming improved grain yield in rice under heat stress. The rice plants were sprayed with distilled water, 50 μM ABA or 10 μM fluridone (FLU) at the 5th d after initial heading stage (AIH) or 12th d after initial heading stage, and then subjected to unstressed or heat stress conditions at 8–14 days after initial heading (AIH) stage or 15–21 days after initial heading stage, respectively. (**A**) Image of rice grain at the mature stage under different treatments. (**B**) Harvest index (HI, %) and (**C**) grain yield per plant (GY, g) were measured at the mature stage. Values are means ± SDs, *n* = *3*. Different letters on the column represent significant differences (*P* < *0.05*) between different treatments based on Duncan’s test

As shown in Fig. 5, heat stress at 8–14 d and 15–21 d AIH stage result in significant yield loss, as shown by the lower harvest index (HI) and grain yield (GY). Pretreatment with exogenous ABA significantly increased HI and GY by 9.1% and 6.6%, 16.5% and 14.4% at 8–14 d and 15–21 d AIH stage, respectively (Fig. 5B, C). Under unstressed conditions, exogenous FLU pretreatment induced decrease in HI and GY (Fig. 5B, C), which may be associated with the lower activity levels of AGPase, SSS, and GBSS and higher activity level of DBE.

### Exogenous ABA pretreatment improved rice milling quality and appearance quality under heat stress

**Fig. 6:**
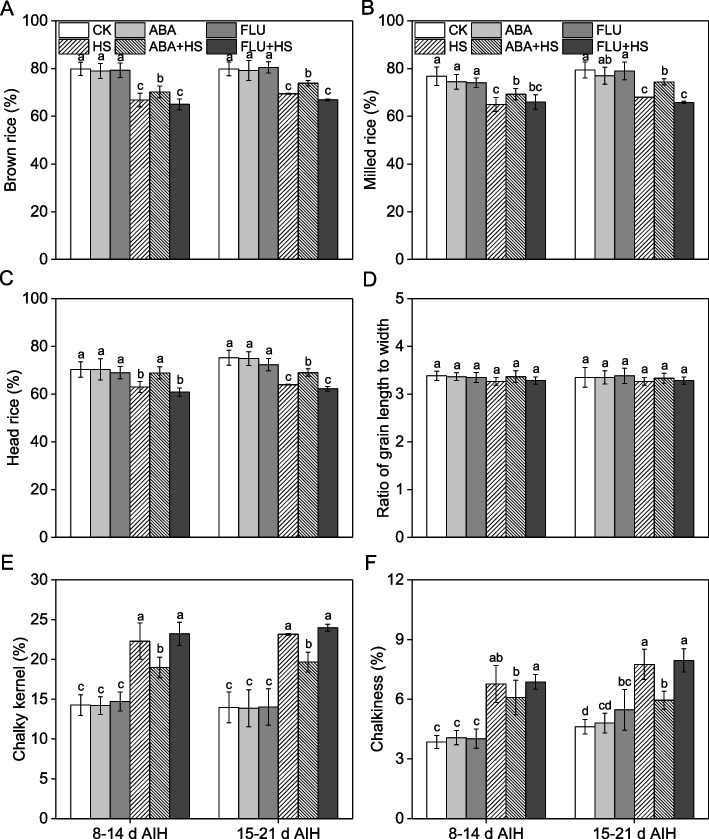
Exogenous ABA priming improved rice appearance and milling quality under heat stress. The rice plants were sprayed with distilled water, 50 μM ABA or 10 μM fluridone (FLU) at the 5th d after initial heading stage (AIH) or 12th d after initial heading stage, and then subjected to unstressed or heat stress conditions at 8–14 days after initial heading (AIH) stage or 15–21 days after initial heading stage, respectively. (**A**) Brown rice, (**B**) milled rice, (**C**) head rice, (D) ratio of grain length to width, (**E**) chalky kernel, and (**F**) chalkiness were measured at the mature stage. Values are means ± SDs, *n* = *3*. Different letters on the column represent significant differences (*P* < *0.05*) between different treatments based on Duncan’s test

As shown in Fig. 6, heat stress at grain filling stage induced a significant decrease in rice milling quality as shown by the lower percentage of brown rice, milled rice, and head rice from the rice grain. In addition, heat stress decreased the quality of appearance by significantly increasing the incidence of chalky kernels and chalkiness in rice grains (Fig. 6). Application of exogenous ABA significantly increased the percentage of brown rice, milled rice, and head rice, and decreased the incidence of chalky kernels and chalkiness under heat stress at different filling stages (Fig. 6). Compared to the HS treatment, exogenous ABA pretreatment increased the percentage of brown rice, milled rice, and head rice by 5.1%, 6.7% and 9.3%, and by 6.3%, 9.4% and 7.9% at 8–14 d and 15–21 d AIH stage, respectively (Fig. 6A-C). The incidence of chalky kernels and chalkiness were decreased by 14.9% and 15.0% and by 10.1% and 23.2% with ABA pretreatment (Fig. 6E, F). There were no significant differences in the ratio of grain length to width between different treatments (Fig. 6B).

### Exogenous ABA pretreatment increased starch content in rice grain under heat stress at the grain filling stage

**Fig. 7:**
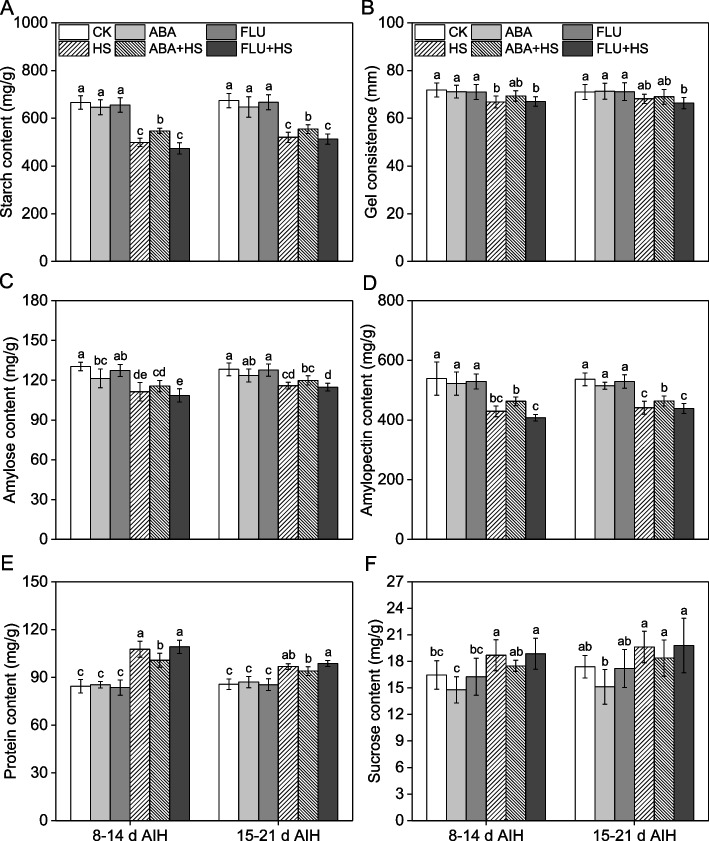
Exogenous ABA priming regulated starch content in rice grain under heat stress. The rice plants were sprayed with distilled water, 50 μM ABA or 10 μM fluridone (FLU) at the 5th d after initial heading stage (AIH) or 12th d after initial heading stage, and then subjected to unstressed or heat stress conditions at 8–14 days after initial heading (AIH) stage or 15–21 days after initial heading stage, respectively. (**A**) Starch content, (**B**) gel consistence, (**C**) amylose content, (**D**) amylopectin content, (**E**) protein content, and (**F**) sucrose content were measured at the mature stage. Values are means ± SDs, *n* = *3*. Different letters on the column represent significant differences (*P* < *0.05*) between different treatments based on Duncan’s test

Heat stress at the grain filling stage caused a significant decrease in the rice grain starch content, as well as the amylose content, amylopectin content and gel consistence (Fig. 7). However, heat stress at the grain filling stage increased the protein and sucrose content of the rice grains. Under heat stress conditions, exogenous ABA pretreatment increased the starch content by 9.6% and 6.7% at 8–14 d and 15–21 d AIH stage, respectively (Fig. 7A). Consistently, compared to the HS treatment, exogenous ABA pretreatment significantly increased the amylose content, amylopectin content and gel consistence by 3.4–3.8%, 5.1–7.9% and 1.2–3.7% under heat stress at 8–14 d and 15–21 d AIH stage (Fig. 7C, D). However, ABA pretreatment decreased the amylose and amylopectin percentages by 3.1–5.3% and 1.6–1.7%, respectively, under heat stress at 8–14 d and 15–21 d AIH stage (Fig. S3). The protein and sucrose contents decreased by 2.9–6.4% and 6.3–6.4%, respectively, with exogenous ABA pretreatment under heat stress at 8–14 d and 15–21 d AIH stage (Fig. 7E, F).

### Effect of exogenous paraquat and antioxidant (proanthocyanidins, PCs) on ROS content, starch synthetase activities, yield component and starch content under heat stress conditions

**Fig. 8:**
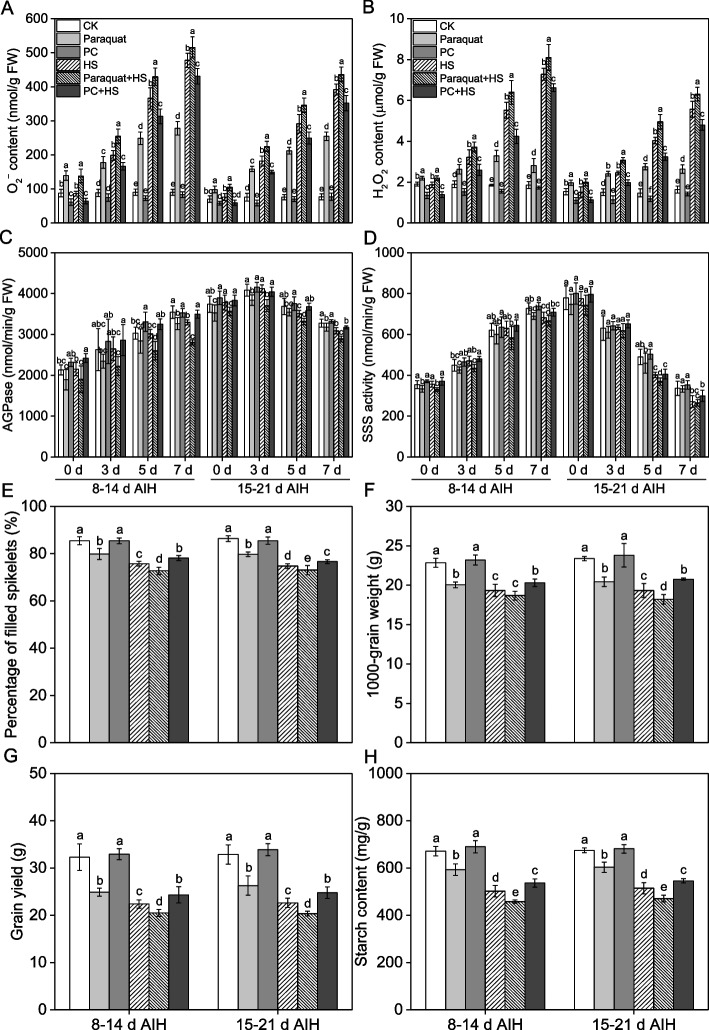
Effect of exogenous paraquat and antioxidant (proanthocyanidins, PCs) on ROS content, starch synthetase activities, yield component and starch content in rice under heat stress conditions. The rice plants were sprayed with distilled water, 10 μM paraquat or 1% PCs at the 5th d after initial heading stage (AIH) or 12th d after initial heading stage, and then subjected to unstressed or heat stress conditions at 8–14 days after initial heading (AIH) stage or 15–21 days after initial heading stage, respectively. Rice grains at the indicated treatment times of 0 d, 3 d, 5 d and 7 d were sampled in liquid nitrogen which were used to measure the ROS contents and starch synthetase activities. The levels of O_2_^–^ (**A**) and H_2_O_2_ (**B**) and the activity levels of AGPase (**C**) and SSS (**D**) in rice grains were measured at the indicated treatment days. (**E**) Percentage of filled spikelets (PFS, %), (**F**) 1000-grain weight (TGW, g), (**G**) grain yield per plant (GY, g) and (**H**) starch content were measured at the mature stage. Values are means ± SDs, *n* = *3*. Different letters on the column represent significant differences (*P* < *0.05*) between different treatments at the same treatment day based on Duncan’s test

Exogenous paraquat and antioxidants (proanthocyanidins, PCs) were used to investigate the relationship between ROS accumulation and starch synthesis capacity under heat stress. As shown in Fig. 8, application of exogenous paraquat caused overaccumulation of ROS as shown by higher levels of O_2_^–^ and H_2_O_2_, while application of PCs significantly decreased ROS accumulation in the rice grain. Compared to the HS treatment, paraquat treatment increased the O_2_^–^ content by 7.9–59.8% and 11.3–37.2%, and the H_2_O_2_ content was increased by 11.3–16.1% and 13.3–32.7% at 8–14 d and 15–21 d AIH stage, respectively (Fig. 8A, B). Compared to the HS treatment, the PC treatment decreased the levels of O_2_^–^ and H_2_O_2_ by 9.8–25.5% and 9.0–25.9% under heat stress conditions (Fig. 8A, B).

Compared to the HS treatment, application of paraquat significantly decreased the activity of AGPase as shown by the decreased levels of 11.4–15.9% and 5.4–10.2% at 8–14 d and 15–21 d AIH stage, respectively (Fig. 8C). The decreased level of SSS activity was by 2.3–7.4% and 2.2–8.0% with paraquat treatment compared to HS treatment at 8–14 d and 15–21 d AIH stage, respectively (Fig. 8D). Compared to the HS treatment, application of PCs increased the AGPase and SSS activity levels by 2.7–12.5% and 2.1–10.4% at 8–14 d and 15–21 d AIH stage (Fig. 8C, D).

Furthermore, the application of paraquat caused significant decrease in the yield component index of percentage of filled spikelets (PFS) and 1000-grain weight (TGW) (Fig. 8E, F). Compared to the HS treatment, paraquat treatment decreased the PFS and TGW by 2.3–4.0% and 3.4–5.6%, while PC treatment increased PFS and TGW by 2.5–3.2% and 5.1–7.5% under heat stress (Fig. 8E, F). Compared to the HS treatment, paraquat treatment decreased the final yield by 8.4–10.1% and PC treatment increased the yield by 8.7–9.5% under heat stress conditions (Fig. 8G). Compared to the HS treatment, the paraquat treatment decreased the starch content in rice grain by 8.8% and 8.5% and PC treatment increased the starch content in rice grain by 6.8% and 6.1% under heat stress conditions (Fig. 8H). In addition, application of paraquat caused significant decrease in the ratio of brown rice and milled rice, and significant increase in the incidence of chalky kernels and chalkiness in rice grain (Fig. S4). On the contrary, PC treatment increased the ratio of brown rice and milled rice, and decreased the incidence of chalky kernels and chalkiness in rice grain (Fig. S4).

## Discussion

Heat stress has become an enormous meteorological disaster for crop production and is characterized by an extreme or lasting high-temperature climate that persists for a long time [1]. Rice is the most important food crop worldwide, and rice production suffers severe heat damage during different growth stage [28]. Rice is more sensitive to heat stress during the reproductive growth stage. Heat stress at the booting stage leads to growth inhibition in the young panicle and decreases panicle length or weight in rice [5]. Heat stress has been found to reduce the percentage of filled spikelets in rice at the heading stage as reflected in decreased pollen viability, disrupted meiosis and pollen abortion [9, 10, 29]. At the grain-filling stage, heat stress inhibits starch synthesis capacity and decreases spikelet weight, which ultimately leads to severe yield loss and decline in rice quality [17, 30]. Phytohormones, such as salicylic acid, kinetin and ABA, play an important role in crop response to heat stress, [20, 31]. We previously reported that ABA primes rice for enhanced alkali stress tolerance and yield in saline-alkaline paddy soils [24, 25, 32, 33]. Pretreatment with exogenous ABA also improved rice tolerance to heat stress by means of seed soaking, root drenching and leaf spraying [6, 8, 10]. In the present study, rice plants pretreated with ABA had significantly upregulated expression levels of ABA responsive genes (Fig. 1), indicating that the ABA signal pathway was remarkably activated under heat stress conditions. ABA pretreatment significantly improved antioxidant defense capacity (Fig. 2, S1), decreased ROS content (Fig. 2) and improved starch synthetase activities (Fig. 3) under heat stress conditions. Finally, ABA pretreatment alleviated yield loss (Figs. 4, 5), and improved rice quality (Fig. 6) and starch synthesis capacity (Fig. 7, S3) under heat stress at the grain filling stage. Application of fluridone inhibited the expression of ABA responsive genes, and caused the overaccumulation of ROS and severe yield loss in rice grain under heat stress conditions. These data suggest that exogenous ABA pretreatment has a potential priming effect for enhancing heat stress tolerance in rice at grain filling stage via the upregulation of antioxidant defense capacity. In addition, application of paraquat caused excess ROS accumulation in rice grain, and then decreased starch synthetase activities in rice grain and resulted in severe yield loss under heat stress (Fig. 8). However, the exogenous antioxidant PCs significantly decreased the ROS content and improved yield component under heat stress (Fig. 8). These results showed that decreasing of ROS content contributed to improve starch synthesis capacity under heat stress condition for maintaining favorable grain filling process. In a word, these data in the present study collectively suggest that pretreatment with exogenous ABA at the grain filling stage primes rice grain for improving ROS-scavenging capability, mitigating oxidative stress induced by ROS overaccumulation and enhancing starch synthesis capacity, so as to improve grain yield and rice quality under heat stress conditions.

The phytohormone ABA plays vital roles in the regulation of plant growth and in coping with various environmental stress factors [22, 24]. The priming effect is an important mechanism by which ABA enhances stress tolerance in plants, which helps plants acquire the potential ability to enhance their adaptive capacity response to subsequent stress factors [23, 33]. The priming effect of ABA has been validated in rice responses to saline and alkaline stress both under greenhouse and field conditions [24, 32]. ABA also functions in the rice response to heat stress [27]. We previously reported the priming effect of ABA on rice growth and yield formation at the germination, seedling and heading stages [6, 8, 10]. In the present study, two ABA responsive genes, *SalT* and *OsWsi18* (Fig. 1) were significantly upregulated by heat stress, which indicated that ABA signaling was activated in the rice response to heat stress. However, grain yield was markedly decreased by heat stress, as well as rice quality, which indicated that this activation of ABA signaling was not effective for rice to cope with heat stress. Nevertheless, ABA pretreatment by leaf and panicle spraying super-induced the expression of ABA responsive genes (Fig. 1), as well as the ROS-scavenging capacity (Fig. 2, S1), to inhibit the overaccumulation of ROS (Fig. 2) in the rice grain, and improved starch synthetase activities (Fig. 3), which finally alleviated yield loss (Fig. 4, 5) and decline of rice quality (Fig. 6, 7), under heat stress conditions. The application of ABA synthesis inhibitor, fluridone, significantly suppressed the expression of ABA responsive genes, decreased the activity levels of antioxidative enzymes and caused the overaccumulation of ROS, ultimately resulting in severe yield loss under heat stress at the grain filling stage, which showed contrasting effects on ABA application. These results were in alignment with the findings of previous studies at the seedling and heading stages [8, 10], which suggested that exogenous ABA enhances tolerance to heat stress in rice plants at the grain filling stage via the priming effect.

ROS act as important messengers in plant growth and regulation of plant responses to various stress factors [34]. However, overaccumulation of ROS induced by various stress factors causes severe damage to plants [14]. Excess accumulation of ROS in rice roots induced by alkaline stress was a key causal factor for cell damage and root injury in rice seedlings [16]. Under heat stress conditions, the overaccumulation of ROS causes damage to the membrane and pollen viability [17], inhibits young bud [6], withers the leaves of rice seedlings [8] and decreases the percentage of filled spikelets [10]. In the present study, under unstressed condition, ABA pretreatment induced ROS accumulation in rice grain, indicating the synergistic effect between ABA and ROS in the regulation of plant growth [17, 35]. Application of ABA would increase ROS content in plants [36, 37], but this increasing range was less than the stress factors-induced ROS increasing range [8, 24]. Results of the present study showed that heat stress caused excess accumulation of ROS in the rice grain at the grain filling stage as shown by a gradually rising accumulation of O_2_^–^ and H_2_O_2_ in grains at the indicated time (Fig. 2A, B). Meanwhile, the activity levels of antioxidant defense enzymes were increased by heat stress (Fig. 2C-F), and the ROS-scavenging genes were upregulated (Fig. S1). These data indicated that the ROS signaling pathway was induced by heat stress in rice at the grain filling stage. However, the antioxidant defense system was damaged by severe heat stress factor which sustained for long times as shown by the decline of activities in SOD and POD at 7 d of heat stress (Fig. 3). In addition, the ROS content was too high in turn, leading to decrease in the yield component (Figs. 4 and 5) and rice quality indices (Fig. 6) under heat stress. The application of paraquat resulted in excess ROS accumulation in rice grain (Fig. 8A, B), decreased the starch synthetase activities (Fig. 8A, B), and caused significant decrease in the yield component (Fig. 8E-G), starch content (Fig. 8H) and rice quality indices (Fig. S4). In contrast, the application of PCs resulted in significant scavenging of the over-accumulated ROS to improved starch synthetase activities, grain yield and rice quality (Fig. 8, S4). These data demonstrated another restrictive factor in ROS inhibiting yield formation in rice grain, which referred to that the yield loss and decline in rice quality caused by heat stress at the grain filling stage were associated with the decline in starch synthetase activities caused by overaccumulation of ROS in rice grains. Therefore, it is conceivable that inhibiting ROS producing rate or strengthening ROS-scavenging capacity by the molecular engineering technologies may help to improve rice productivity and quality under heat stress condition. This result was in accordance with the findings of previous studies [10, 17, 19].

Grain filling is a process of starch synthesis and accumulation in rice grain [38]. The synthesis of starch in rice grain is regulated by various metabolic enzymes, such as ADP-glucose pyrophosphorylase (AGPase), soluble starch synthase (SSS), granule-bound starch synthase (GBSS), starch branching enzyme (SBE), starch debranching enzyme (DEB) and sucrose synthase (SS) [5, 11]. Heat stress influences starch synthesis in rice grain by affecting these starch synthesis-related enzymes [39]. In the present study, the activity levels of starch synthesis-related enzymes were little affected by short-time heat stress factor, while continuous heat stress affected the metabolic activity of these enzymes which may be due to the overaccumulation of ROS [40, 41]. Pretreatment with ABA increased the AGPase, SSS, GBSS and SS activity levels and decreased the SBE and DBE activity levels (Fig. 3). This indicated that ABA possibly functions the starch synthesis process by increasing starch synthesis capacity and decreasing the transformation or metabolism rate to improve grain filling capacity. In addition, the application of paraquat significantly decreased the starch synthetase activities and starch content (Fig. 8), while exogenous antioxidant PCs improved the starch synthesis capacity and starch content in rice grain (Fig. 8). These data suggested that ABA pretreatment improved starch synthesis capacity under heat stress, which may be associated with the upregulation of antioxidative defense capability. ABA functions gene expression network in rice under various stress factors. ABA pretreatment regulated the stress tolerance-related genes, such as cell defense and Na^+^/K^+^ transport genes for participation in the response to alkaline stress in rice seedlings [24]. Under heat stress condition, priming effect of ABA in rice seedlings was associated with the upregulation of heat shock-related genes [8]. In the present study, another mechanism of ABA improving starch synthesis-related enzymes activities may be associated with the regulation in genes expression by ABA. However, the molecular regulatory mechanism of ABA in starch synthesis-related enzymes activities in rice requires a sophisticated experimental design to address this issue.

Previous studies reported that inhibiting ROS overaccumulation was an important mechanism for ABA primes plant enhancing tolerance to stress factors [8, 24]. Under alkaline stress conditions, alleviating cell damage, decreasing numbers of dead cell and then mitigating root injury induced by ROS overaccumulation in roots was a main pathway of ABA pretreatment enhancing alkaline stress tolerance in rice [16, 24, 25]. Under heat stress conditions, ABA also decreased ROS accumulation in rice young bud, leaf and grain for alleviating membrane peroxidation and cell injury, and then contributed to improve growth of plumule and seedlings, and mitigate damage of reproductive organs at different growth stages [6, 8, 10, 41]. Therefore, ROS accumulation levels could be used as the reference indices in the evaluation of heat stress tolerant rice varieties at heading stage [42]. At the grain filling stage, ROS overaccumulation induced by heat stress results in the decrease of rice yield and increase of chalkiness [17, 43]. In the present study, the application of paraquat induced excess ROS accumulation in the rice grain and decreased activity levels of starch synthesis-related enzymes, and then decreased the final rice yield and quality. Enhancing ROS-scavenging capacity could increase starch synthesis enzymes activities and then improve rice quality. Furthermore, ABA pretreatment remarkably decreased ROS accumulation in rice under heat stress. These data collectively suggest ABA enhanced grain filling capacity in rice via upregulating antioxidative defense system and inhibiting ROS overaccumulation to improve starch synthesis enzymes activity levels in the rice grain under heat stress conditions.

In summary, in this study, ABA priming super-increased the ABA signal in rice under heat stress, to upregulate antioxidative defense capacity and decrease ROS content in rice grain for improving starch synthesis activities, and finally increased grain filling capacity at grain filling stage.

## Materials and methods

### Plant material and growth conditions

According to the methods of our previous studies [8, 10], rice cultivar Huanghuazhan, an elite cultivar suitable for widespread in South China, was used in this study. It was bred by crossing the cultivars ‘Huangxinzhan’ with ‘Fenghuazhan’ and was found to be resistant to heat stress (China Rice Data Center). Rice seeds were surface-sterilized with 75% (v/v) alcohol for 5 min and rinsed with deionized water five times. Next, the seeds were immersed in water for 2 days and then sprinkled onto wet filter paper in a petri dish for pre-germination. The uniformly germinated seeds were sown at a nursery site with humus soil that contained 30.9 g/kg organic matter, 2.3 g/kg total nitrogen, 20.6 mg/kg readily available phosphorus, and 142.4 mg/kg readily available potassium, respectively [10]. Healthy rice seedlings were transplanted into pots at the three-leaf stage. The height of the pots was 28 cm, and the internal diameter was 25 cm. Each pot contained 10 kg of experimental paddy soil. The paddy soil contained organic matter at 26.3 g/kg, total nitrogen at 1.9 g/kg, readily available phosphorus at 15.3 mg/kg, readily available potassium at 106.8 mg/kg [10]. The experimental paddy soil was pounded, sifted, dried and then mixed before being placed into the pots. According to a previously published fertilization method [44], 4.5 g of compound fertilizer, in which the levels of N, P, and K were all 15%, was applied to each experimental paddy soil pot, and 3.0 g of urea was applied to each pot at the tillering stage for the after manuring. Two clumps with 3 rice seedlings per clump were transplanted into each pot. Rice plants were cultivated to the initial heading stage under normal conditions with traditional cultivation management methods.

### ABA pretreatment and heat stress treatment

Abscisic acid (ABA) (Sigma, Inc., St, Louis, MO, USA) was dissolved in a small amount of absolute ethanol and then diluted with deionized water to the desired concentrations [24]. According to the methods of our previous study [10], 50 μM ABA and 10 μM fluridone (FLU), an ABA biosynthesis inhibitor, were used in this study. The rice plants at the 5th d after initial heading (AIH) stage and 12th d after initial heading stage were pretreated with 50 μM ABA, 10 μM FLU and deionized water by leaf and panicle spraying for three days, respectively. Then these three sets of rice plants were transferred to the control and heat stress conditions for seven days at the aforementioned two growth stages, which represented as 8–14 d after initial heading stage (8–14 d AIH) and 15–21 d after initial heading stage (15–21 d AIH), respectively. Thus, six treatments were applied during each growth stage: spraying with deionized water under unstressed conditions (CK); spraying with ABA under unstressed conditions (ABA); spraying with FLU under unstressed conditions (FLU); spraying with deionized water under heat stress conditions (HS), spraying with ABA under heat stress conditions (ABA + HS); and spraying with FLU under heat stress conditions (FLU + HS). According to the previous studies on the growth temperature of rice response to heat stress in South China [2, 3, 10], the temperature for the unstressed conditions were set as 32 ℃ day time (7:00–19:00)/26 ℃ night time (19:00–7:00), and the temperatures for the heat stress conditions were set as 38 ℃ from 9:00 to 16:00 and 30 ℃ at other times. All the experiments were conducted in controlled growth chambers under the following conditions: 12-h photoperiods, 350 μmol photons m^−2^ s^−1^ light intensity, and 80% relative humidity. After heat stress treatment, the rice plants were transferred to normal conditions to cultivate to the mature stage.

### Treatment of rice plants with exogenous paraquat and proanthocyanidins (PCs)

In this study, exogenous paraquat and proanthocyanidins (PCs) were used to examine the relationship between ROS levels and starch synthesis capability. Paraquat is a well-established ROS generator that has been extensively used in plant science studies [45]. It induces the production of superoxide radicals, which serve as a source for generating other ROS such as H_2_O_2_ and hydroxyl radicals [46]. Previous study has showed that paraquat caused withering and death in rice seedlings owing to the formation of ROS [24, 45]. PCs are a class of natural polyphenolic antioxidants that have been demonstrated to effectively scavenge ROS and alleviate heat stress-induced suppression of rice plant growth by inhibiting ROS overaccumulation [8, 10]. According to the methods of previous studies, 10 μM paraquat and 1% PCs were used in this study [8, 10, 24]. The rice plants were pretreated with deionized water, 10 μM paraquat, and 1% PCs, by leaf and panicle spraying for three days, respectively; and then were transferred to the aforementioned control or heat stress conditions. The treatments were represented as follows: CK, paraquat, PC, HS, paraquat + HS and PC + HS.

### Measurement of plant growth, grain yield and yield components

At the mature stage, all rice plants were harvested to determine the following parameters: shoot length (SL), shoot dry weight (SDW), primary branches (PB) and secondary branches (SB) of panicle, panicle length (PL), panicle weight (PW), yield components including panicle numbers (PN), spikelets per panicle (SP), numbers and weight of filled spikelets (FS) and empty spikelets (ES), percentage of filled spikelets (PFS), 1000-grain weight (TGW), harvest index (HI) and grain yield (GY). All panicles and spikelets from an individual plant were measured and averaged. Grain yield and yield components were measured as described by Yoshida [47]. The TGW and aboveground biomass were adjusted to 0.14 g/g moisture content on a dry weight basis. HI was calculated as grain yield divided by aboveground biomass. Photographs of the grain yield of different treatments were taken at the mature stage.

### Measurement of rice grain appearance and milling indices

According to the methods described in the National Standard of the People's Republic of China (GB/T17891-1999, high quality paddy), brown rice, milled rice and head rice were measured for the rice grain. Then the milled rice was used to measure the ratio of grain length to width, the incidence of chalky kernels, and the chalkiness according to the aforementioned National Standard aforementioned [13].

### Measurement of starch, amylose, amylopectin, protein and sucrose content and gel consistence

The starch content was measured according to the description by López-Delgado et al. [48]. The 80% ethanol reagent (v/v) was used to separate the soluble sugar and starch in samples. Furthermore, the starch was decomposed to glucose by the acid hydrolysis method and the glucose content was determined by anthrone colorimetry [49]. According to the equation of starch hydrolyzing to glucose, a standard curve was drawn between the glucose concentration and absorbance value of starch content. Finally, the starch content in the samples was calculated according to the glucose concentration [48].

To measure the levels of amylose and amylopectin, amylose and amylopectin were extracted from the milled rice by 80% ethanol reagent (v/v) and diethyl ether. The potassium hydroxide solution was added to dissolve the sediment which contained amylose and amylopectin, and then potassium iodide-iodine reagent was added. The absorbances at 550, 620 and 743 nm were recorded to calculate the content and percentage of amylose and amylopectin [50].

The BCA (bicinchoninic acid) method was used to measure the protein content [51]. Distilled water was added to the milled rice sample and centrifuged at 12,000 × g for 10 min at 4 ℃. The liquid supernatant was extracted for further testing. The BCA reagent, sodium hydroxide solution and sodium tartrate were added into the liquid supernatant. After mixing, the copper sulfate solution was added and warmed at 60 ℃ for 30 min. The absorbances at 562 nm were recorded to calculate the protein content.

According to the description by Nielsen et al. [52], the sucrose content was measured using the resorcinol method. Ethyl alcohol was added to the milled rice sample, ground and centrifuged at 4000 × g for 10 min at 25 ℃. Then the sodium hydroxide solution was added into the liquid supernatant to break the reducing sugar. Then, hydrochloric acid and resorcinol were added to the test sample and warmed at 95 ℃ for 30 min. The absorbances at 480 nm were recorded to calculate the sucrose content.

The gel consistence in milled rice was measured according to the methods described in the National Standard of the People's Republic of China (GB/T17891-1999, high quality paddy) [13].

### Measurement of ROS levels

Rice grain at 0, 3, 5, and 7 d during the heat stress process was sampled in liquid nitrogen for the measurement of ROS levels, antioxidant enzyme activity levels and starch synthetase-related enzyme activity levels.

The O_2_^–^ content was measured as described by Elstner and Heupel [53] by monitoring nitrite formation from hydroxylamine in the presence of O_2_^–^, with some modifications as described by Liu et al. [8]. The absorbance values for the aqueous solution were read at 530 nm to calculate the levels of O_2_^–^ from the chemical reaction of O_2_^–^ and hydroxylamine.

The H_2_O_2_ content was measured by monitoring the A_415_ of the titanium-peroxide complex as previously described [54]. According to the methods of our previous study, the absorbance values in the aqueous solution were read at 415 nm to calculate the levels of H_2_O_2_ [8].

The analytical reagent used to measure the H_2_O_2_ and O_2_^–^ contents were acquired from the determination kit, according to the manufacturer's instructions (Suzhou Michy Biomedical Technology Co., Ltd. China) [8, 10].

### Measurement of antioxidant enzyme activities

According to previous experimental methods, the activity levels of SOD, POD, CAT, and APX were measured using a UV–visible spectrophotometer (UV-2700, Shimadzu, Kyoto, Japan) at the relevant wavelength [24]. SOD (EC 1.15.1.1) activity was determined by the nitro blue tetrazolium (NBT) method as described by Giannopolitis and Ries [55]. One unit of SOD activity was defined as amount of enzyme required to cause 50% inhibition of NBT reduction, as monitored at 560 nm. POD (EC 1.11.1.7) activity was determined by assaying the rate of guaiacol oxidation in the presence of H_2_O_2_ [56]. One unit of POD activity was defined by the variable quantity in absorbance per minute at 470 nm. CAT (EC 1.11.1.6) activity was determined by the reaction level with H_2_O_2_ per unit time [57]. One unit of CAT activity was defined by the variable quantity in absorbance per minute at 240 nm. APX (EC 1.11.1.11) activity was determined using the rate of ascorbic acid (AsA) oxidation by H_2_O_2_ [58]. One unit of APX was defined as the variable quantity of absorbance per minute at 290 nm.

### Measurement of starch synthetase activities

ADP-glucose pyrophosphorylase (AGPase, EC 2.7.7.27) activity was determined by measuring the increase rate of NADPH at 340 nm, as the described by Choix et al. [59]. According to the description by Jiang et al. [39], the activity of soluble starch synthase (SSS, EC 2.4.1.13) and granule-bound starch synthase (GBSS, EC 2.4.1.21) was determined by measuring the generation rate of ADP in the reaction process between ADPG and glucan. The absorbance at 340 nm was recorded to calculate the activity of SSS or GBSS. The starch branching enzyme (SBE, EC 2.4.1.18) was a catalyst for the reaction of amylose transformation to amylopectin at 660 nm. The activity of SBE was determined by the rate of absorbance decrease for the starch-iodine complex at 660 nm [39]. The activity of starch debranching enzyme (DBE, EC 2.4.1.41) was determined by measuring the content of reducing sugar generated by DBE according to the description by Wang et al. [60]. The reducing sugar content was measured by the 3, 5-dinitrosalicylic acid method as described by Rahman et al. [61]. The sucrose synthase (SS, decomposition direction, EC 2.4.1.13) is a catalyst for the decomposition of sucrose to fructose and uridine diphosphate glucose (UDPG) [62]. The SS activity was determined by measuring the reducing sugar content in the abovementioned reaction by the 3, 5-dinitrosalicylic acid method [62].

### RNA isolation and quantitative real-time PCR (qRT-PCR)

Rice grains were sampled in liquid nitrogen and ground using a bench-top ball-mill (Scientz-48, Ningbo Scientz Biotechnology Co. Ltd., Ningbo, China) at 50 Hz for 30 s. Total RNA was extracted with TRIzol reagent (TaKaRa Bio Tokyo, Japan) and first-strand cDNA was synthesized using M-MLV reverse transcriptase (Thermo, Carlsbad, CA, USA) according to the manufacturer’s protocols. Quantitative real-time PCR (qRT-PCR) was performed to determine the transcriptional expression of genes, including two ABA-response genes, *SalT* [24, 63, 64] and *OsWsi18* [24, 64, 65], and eight ROS-scavenging genes, *OsCATA*, *OsCATB*, *OsAPX6*, *OsAPX7*, *OsCu/Zn-SOD*, *OsFe-SOD*, *Putative copper/zinc superoxide dismutase* and *SODCc2* [6]. Gene-specific primers were designed using Primer 5.0 software (Table S1).

The housekeeping gene *β-actin* (GenBank ID: X15865.1) was used as an internal standard. PCR was conducted in a 20 µL reaction mixture containing 1.6 µL of cDNA template (50 ng), 0.4 µL of 10 mM specific forward primer, 0.4 µL of 10 mM specific reverse primer, 10 µL of 2 × SYBR® *Premix Ex Taq*™ (TaKaRa, Bio Inc.), and 7.6 µL of double-distilled H_2_O in a PCR machine (qTOWER2.2. Analytic Jena. GER) [8, 16]. The procedure was performed as follows: 1 cycle for 30 s at 95 °C, 40 cycles for 5 s at 95 °C, and 20 s at 60° C, and 1 cycle for 60 s at 95 °C, 30 s at 55 °C, and 30 s at 95 °C for melting curve analysis [16, 25]. The level of relative expression was computed using the 2^−△△CT^ method [66].

### Experimental design and statistical analyses

All the experiments were performed with three biological replicates. Statistical analyses were performed using the statistical software SPSS 21.0 (IBM Corp., Armonk, NY). Based on a one-way analysis of variance (ANOVA), Duncan’s multiple range test (DMRT) was used to compare differences in the means among treatments. The significance level was *P* < 0.05.

### Supplementary Information

**Additional file 1: Table S1. **Genes and primer sequences used for qRT-PCR. **Fig. S1.** Exogenous ABA priming upregulated the transcriptional expression levels of ROS-scavenging genes in rice grains under heat stress. **Fig. S2.** Effect of exogenous ABA on rice growth under heat stress conditions. **Fig. S3.** Exogenous ABA priming regulated starch content in rice grain under heat stress. **Fig. S4. **Effect of exogenous paraquat and antioxidant (proanthocyanidins, PC) on rice quality of appearance indices under heat stress conditions.

## Data Availability

All data generated or analysed during this study are included in this published article and the supplementary information files.

## Abbreviations

ABA: Abscisic acid
AIH: After initial heading
AGPase: ADP-glucose pyrophosphorylase
DBE: Dtarch debranching enzyme
ES: Empty spikelets
FLU: Fluridone
FS: Filled spikelets
GBSS: Granule-bound starch synthase
GY: Grain yield
H_2_O_2_: Hydrogen peroxide
HI: Harvest index
HS: Heat stress
O_2_^–^: Superoxide anions
PB: Primary branches
PCs: Proanthocyanidins
PFS: Percentage of filled spikelets
PL: Panicle length
PN: Panicle number
PW: Panicle weight
qRT-PCR: Quantitative real-time PCR
ROS: Reactive oxygen species
RBOH: Respiratory Burst Oxidase Homolog
SB: Secondary branches
SBE: Starch branching enzyme
SDW: Shoot dry weight
SL: Shoot length
SP: Spikelets per panicle
SS: Sucrose synthase
SSS: Soluble starch synthase
TWG: 1000-Grain weight
