# Supplementary material for: Exogenous abscisic acid improves grain filling capacity under heat stress by enhancing antioxidative defense capability in rice
Source: BMC Plant Biol. 2023 Dec 6;23:619. doi: 10.1186/s12870-023-04638-5 (PMC10699063; doi:10.1186/s12870-023-04638-5)
Supplement: Supplementary file 1 — Additional file 1: Table S1. Genes and primer sequences used for qRT-PCR. Fig. S1. Exogenous ABA priming upregulated the transcriptional expression levels of ROS-scavenging genes in rice grains under heat stress. Fig. S2. Effect of exogenous ABA on rice growth under heat stress conditions. Fig. S3. Exogenous ABA priming regulated starch content in rice grain under heat stress. Fig. S4. Effect of exogenous paraquat and antioxidant (proanthocyanidins, PC) on rice quality of appearance indices under heat stress conditions. [file 12870_2023_4638_MOESM1_ESM.docx]

**Supplementary material**

**Table S1 Genes and primer sequences used for qRT-PCR.**

| Gene name | RAP-DB ID | Forward (5'→3') | Reverse (5'→3') |
| --- | --- | --- | --- |
| *OsACT1*  *SalT*  *OsWsi18*  *OsCATA*  *OsCATB*  *OsAPX6*  *OsAPX7*  Putative copper/zinc superoxide dismutase  *OsFeSOD*  *SodCc2*  *OsCu/Zn-SOD* | Os03g0718100  Os01t0348900  Os01g0705200  Os02g0115700  Os06g0727200  Os12g0178100  Os04g0434800  Os03g0219200  Os06g0143000  Os07g0665200  Os08g0561700 | TTCCAGCCTTCCTTCATA  CGAAATAATGTTCCATGGTGTT  TGTGACTCGATCCAGCGTAG  CCCCAAGGTCTCCCCTGA  GCTGGTGAGAGATACCGGTCA  CCCCAAGATCCCCATGATCTA  TTCACGTTGGACGGTTAATGC  CAGATTTCACTAAGCGGGCC  CGACGCCGAGGAATTTCTAG  ATTCCATGTGCACGCGC  TGTGACGGGACTTACTCCTGG | AACGATGTTGCCATATAGAT  TGTACTACGGATCGGTGCAA  GTTCCTGCTGAGAAGCCATC  AACGACTCATCACACTGGGAGAG  TCAACCCACCGCTGGAGA  CCTCTGGCGGGCATTG  TTTCTGTAAAAGTGGTTGGCCA  CTTCCTAGGTCATCAGAATCAGCA  AGGTGGTGTAAGTGTCTCTCATGC  GGATTGAAGTGTGGTCCAGTTG  CACCCATTCGTAGTATCGCCA |

**Figure S1**





**Fig. S1** Exogenous ABA priming upregulated the transcriptional expression levels of ROS-scavenging genes in rice grains under heat stress. The rice plants were sprayed with distilled water, 50 μM ABA or 10 μM fluridone (FLU) at the 5th d after initial heading stage (AIH) or 12th d after initial heading stage, and then subjected to unstressed or heat stress conditions at 8-14 days after initial heading (AIH) stage or 15-21 days after initial heading stage, respectively. Rice grains at the indicated treatment time of 5 d were sampled in liquid nitrogen which were used to measure the expression levels of relative genes. Relative expression levels of the ROS-scavenging genes, *OsCATA* (**A**), *OsCATB* (**B**), *OsAPX6* (**C**), *OsAPX7* (**D**), *OsCu/Zn-SOD* (**E**), *OsFe-SOD* (**F**), *Putative copper/zinc superoxide dismutase* (**G**) and *SODCc2* (**H**) were measured at the indicated treatment days. A quantitative real-time polymerase chain reaction was performed using *OsACT1* as an internal standard. The expression levels of the untreated control (CK) at 0 d were set as the unit to calculate the expression levels. Values are means ± SDs, *n=3*. Different letters on the column represent significant differences (*P<0.05*) between different treatments based on Duncan’s test.

**Figure S2**





**Fig. S2** Effect of exogenous ABA on rice growth under heat stress conditions. The rice plants were sprayed with distilled water, 50 μM ABA or 10 μM fluridone (FLU) at the 5th d after initial heading stage (AIH) or 12th d after initial heading stage, and then subjected to unstressed or heat stress conditions at 8-14 days after initial heading (AIH) stage or 15-21 days after initial heading stage, respectively. (**A**) Shoot length (SL), (**B**) shoot dry weight (SDW), (**C**) primary branches (PB), (**D**) secondary branches (SB), (**E**) panicle length (PL), (**F**) panicle weight (PW) were measured at the mature stage. Values are means ± SDs, *n=3*. Different letters on the column represent significant differences (*P<0.05*) between different treatments at the same treatment day based on Duncan’s test.

**Figure S3**





**Fig. S3** Exogenous ABA priming regulated starch content in rice grain under heat stress. The rice plants were sprayed with distilled water, 50 μM ABA or 10 μM fluridone (FLU) at the 5th d after initial heading stage (AIH) or 12th d after initial heading stage, and then subjected to unstressed or heat stress conditions at 8-14 days after initial heading (AIH) stage or 15-21 days after initial heading stage, respectively. (**A**) Amylose percentage, (**B**) amylopectin percentage were measured at the mature stage. Values are means ± SDs, *n=3*. Different letters on the column represent significant differences (*P<0.05*) between different treatments at the same treatment day based on Duncan’s test.

**Figure S4**





**Fig. S4** Effect of exogenous paraquat and antioxidant (proanthocyanidins, PC) on rice quality of appearance indices under heat stress conditions. The rice plants were sprayed with distilled water, 10 μM paraquat or 1% PC at the 5th d after initial heading stage (AIH) or 12th d after initial heading stage, and then subjected to unstressed or heat stress conditions at 8-14 days after initial heading (AIH) stage or 15-21 days after initial heading stage, respectively. (**A**) Brown rice, (**B**) milled rice, (**C**) chalky kernel, (**D**) chalkiness were measured at the mature stage. Values are means ± SDs, *n=3*. Different letters on the column represent significant differences (*P<0.05*) between different treatments at the same treatment day based on Duncan’s test.
